# Barriers to healthcare access and healthcare seeking for childhood illnesses among childbearing women in Burundi

**DOI:** 10.1371/journal.pone.0274547

**Published:** 2022-09-30

**Authors:** Bright Opoku Ahinkorah, Abdul-Aziz Seidu, John Elvis Hagan, Eugene Budu, Aliu Mohammed, Collins Adu, Edward Kwabena Ameyaw, Faustina Adoboi, Thomas Schack

**Affiliations:** 1 Faculty of Health, School of Public Health, University of Technology Sydney, Ultimo, Australia; 2 Department of Population and Health, University of Cape Coast, Cape Coast, Ghana; 3 College of Public Health, Medical and Veterinary Sciences, James Cook University, Townsville, Queensland, Australia; 4 Department of Health, Physical Education, and Recreation, University of Cape Coast, Cape Coast, Ghana; 5 Faculty of Psychology and Sport Sciences, Neurocognition and Action-Biomechanics-Research Group, Bielefeld University, Bielefeld, Germany; 6 Department of Health Promotion and Disability Studies, Kwame Nkrumah University of Science and Technology, Kumasi, Ghana; 7 Cape Coast Nursing and Midwifery Training College, Cape Coast, Ghana; University of Washington, UNITED STATES

## Abstract

**Introduction:**

Poor health seeking behaviour continues to be major challenge in accessing healthcare in sub-Saharan Africa despite the availability of effective treatment for most childhood illnesses. The current study investigated the barriers to healthcare access and health seeking for childhood illnesses in Burundi.

**Methods:**

The study utilized data from the 2016–17 Burundi Demographic and Health Survey (BDHS). A total of 2173 children under five of childbearing women were included in our study. The outcome variable for the study was healthcare seeking for childhood illnesses (diarrhea and fever/cough). Barriers to healthcare access were the explanatory variables and maternal and child factors were the control variables. Chi-square test of independence and a binary logistic regression modelling were carried out to generate the results.

**Results:**

Overall, less than 50% of children in Burundi who were ill two weeks before the survey obtained healthcare. We found that children of mothers who perceived getting money for medical care for self as a big problem [aOR = 0.75; CI = 0.60–0.93] and considered going for medical care alone as a big problem [aOR = 0.71; CI = 0.55–0.91] had lower odds of getting healthcare, compared to those of mothers who considered these indicators as not a big problem. The results also showed that children of mothers who had three [aOR = 1.48; 1.02–2.15] and four [aOR = 1.62; 1.10–2.39], children were more likely to get healthcare for childhood illnesses compared to those whose mothers had one child. Children of mothers with single birth children were less likely to get healthcare compared to those whose mothers had multiple births.

**Conclusion:**

Findings of the low prevalence of healthcare for childhood illnesses in Burundi suggest the need for government and non-governmental health organizations to strengthen women’s healthcare accessibility for child healthcare services and health seeking behaviours. The Burundian government through multi-sectoral partnership should strengthen health systems for maternal health and address structural determinants of women’s health by creating favourable conditions to improve the status of women and foster their overall socioeconomic well-being. Free child healthcare policies in Burundi should be strengthened to enhance the utilization of child healthcare services in Burundi.

## Introduction

Despite the tremendous progress made in reducing childhood morbidity and mortality globally, the situation in sub-Saharan Africa (SSA) still remains a major public health concern. For instance, in 2018, the UN Inter-agency Group for Child Mortality Estimation (UN IGME) estimated that 3.3 million children under aged 15 years died in SSA, representing 53% of childhood deaths globally (6.2 million) in the world [1]. Averagely, 1 in 13 children died in SSA compared to 1 in 199 in high income countries in 2019, with most of these childhood deaths (85%) occurring in the first years of life [1,2]. Thus, under-five morbidity and mortality continue to be major public health challenges in SSA. Although under-five mortality rates in Burundi have declined significantly from 174 deaths per 1,000 live births in 1990 to 58 deaths per 1,000 live births in 2018 [1], the rate still remains high, considering the global target of reducing under five mortality to 25 per 1000 live births by 2030 through SDG-3.2.

Childhood deaths are often caused by treatable or preventable diseases such as diarrhoea, malaria, pneumonia, and preterm birth complications [1,2]. In 2010, approximately 93% of all hospitalisation of children between 1 to 59 months in Burundi were due to malaria, respiratory tract infections, and acute diarrhoea [3]. Meanwhile, available evidence suggests that inadequate access to healthcare services [4,5] and inappropriate health seeking behaviour [6] significantly account for most childhood illnesses and resultant deaths in SSA. According to UN IGME [1], about 50% of under-five mortality could be prevented by having timely access to quality healthcare. Access to healthcare continue to be a major challenge in Burundi as the country is ranked 186 out of 195 countries who had best access to quality healthcare in 2016 despite the existence of free child healthcare policies in the country [4].

Accessing healthcare for childhood illnesses is not only determined by distance to healthcare facilities and cost of obtaining healthcare services [5,7], but also the health seeking behaviours of parents/guardians, most notably the mother [8,9]. Akinyemi et al. [10] suggested that poor health seeking behaviour continues to be a major challenge in accessing healthcare in SSA despite the availability of effective treatment for most childhood illnesses. Studies have shown that the decision to seek healthcare for a sick child in SSA is largely determined by the child’s age [11–13], mother’s educational level and marital status [7], mother’s age [14], family size, previous experience of similar illness, history of under-five mortality [12], mother’s knowledge level on danger signs of childhood illnesses such as fever [11], family income [9,15], geographic location or distance [7,11], and ethnicity [8,16]. For example, perceived non-seriousness of a child’s illness delayed or prevented mothers from seeking healthcare services for their children in Ethiopia [17]. Also, parents/guardians in Kenya, who lived more than a kilometre from a health facility, were less likely to seek care for their sick children [7].

Considering the importance of timely access to healthcare and appropriate health seeking behaviour in reducing the severity of childhood illnesses and associated deaths, it is important to investigate the specific barriers to healthcare access and health seeking behaviour, to develop specific strategies that could address them. Therefore, this current study investigated the barriers to healthcare access and health seeking behaviour for childhood illnesses in Burundi. Findings from the study would help make recommendations that could potentially address these barriers and improve childhood morbidity and mortality in the country.

## Materials and methods

### Data source and study design

**Fig 1:**
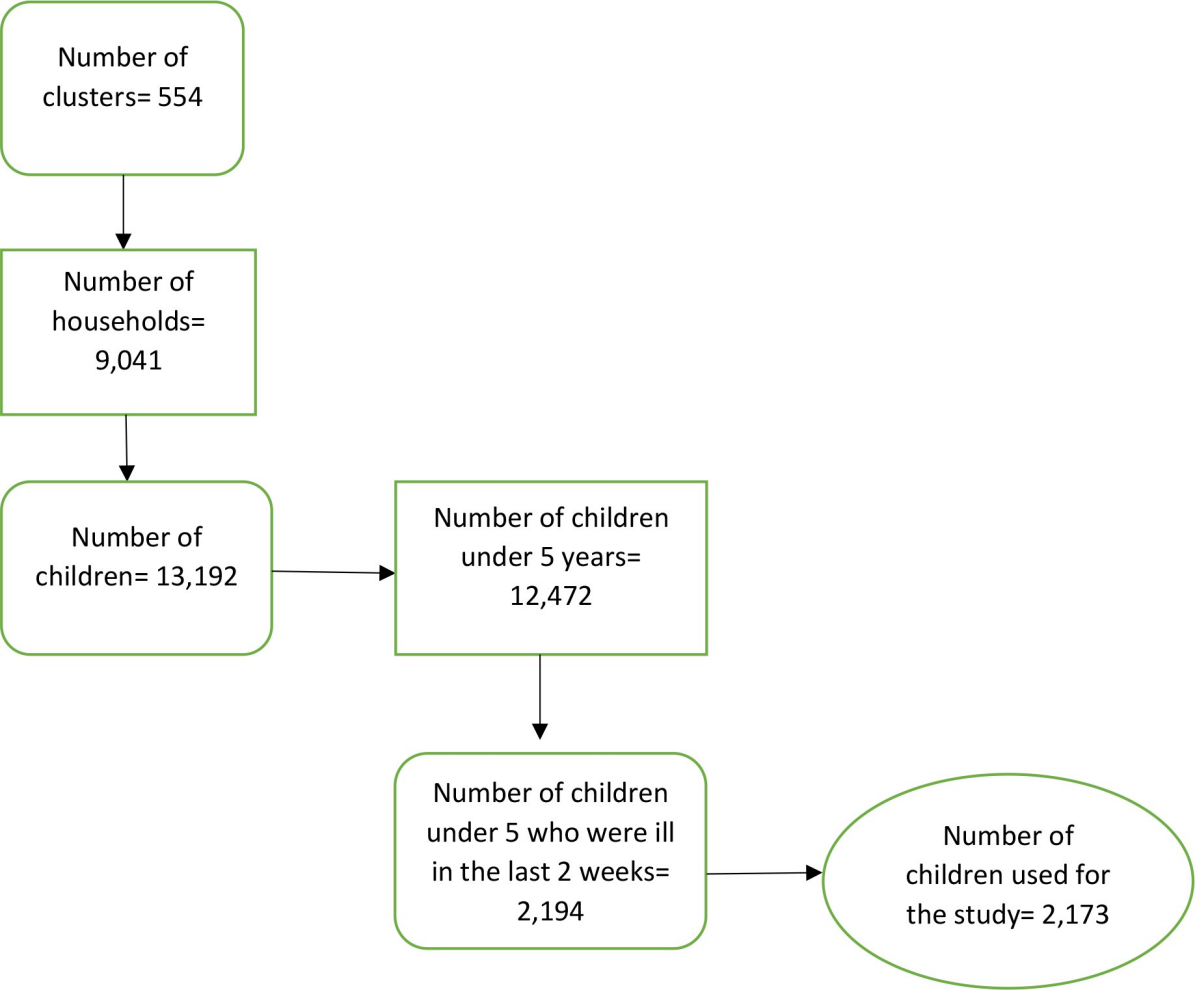
Flowchart showing the sample.

The study employed a cross-sectional study design and used data from the 2016–17 Burundi Demographic and Health Survey (DHS). Specifically, data from the birth recode file, which has one record for every child ever born to interviewed women was used. The DHS is a nationally representative survey that is conducted in over 85 low-and middle-income countries globally. The survey focuses on essential maternal and child health markers including “health seeking behaviour” [18]. The study by Aliaga and Ruilin [19] provides details of the sampling process. The surveys employ a two-stage stratified sampling technique, which makes the survey data nationally representative [19]. The first stage involves the generation of a sampling frame from enumeration areas (EAs) that covered the given country. The EAs are mostly generated from the most recent national census data in the country. Each EA is subsequently segmented into standard size segments of about 100–500 households per segment. The second stage involves a systematic selection of households from the EAs and an in-person interviews in selected households with the various target populations: women (15–49) and men (15–64). The number of selected households per EA ranged from 30 to 40 households/women per rural cluster and from 20 to 25 households/women per urban cluster. A total of 2173 children under five of childbearing women who had complete information on all the variables of interest were included in our study. Since the authors used a secondary data, they were not directly involved in the data collection. However, data collection was done by trained field staff who were responsible for data collection for the survey in Burundi. Fig 1 shows how we arrived at the sample.

### Definition of variables

#### Outcome variable

The outcome variable for the study was health seeking behaviour for childhood illnesses. It was derived as a composite variable from two questions, “Did [NAME] receive treatment for diarrhea?”, and “Did [NAME] receive treatment for fever/cough?” The responses were “yes” and “no”. Women whose children suffered from either diarrhea or fever/cough two weeks prior to the survey responded to these questions. Women who responded that they sought healthcare for either treatment for diarrhea or fever/cough or both were considered as seeking healthcare for childhood illnesses and were given the code 1 = yes while those who responded that they neither sought for treatment for diarrhea nor fever/cough were considered as those who never sought healthcare for childhood illnesses and were coded as 0 = no.

#### Explanatory variables

The study looked at barriers in accessing healthcare as the explanatory variable. In the DHS, barriers in accessing healthcare was generated by asking women if they had serious problems in accessing healthcare for themselves when they are sick. The problems were difficulty with distance to the facility, difficulty in getting money for treatment, difficulty with getting permission to visit health facility, and difficulty in not wanting to go for medical help alone. For each of these questions, the responses were ‘big problem’ and ‘not a big problem’. Although these indicators are asked of women and are not linked to healthcare seeking for the child, we consider these indicators as proxy for accessing barriers women go through when seeking healthcare for the child.

#### Covariates

Fourteen variables were considered in the study as covariates. The variables were age, marital status, employment status, parity, religion, exposure to mass media (radio, television and newspaper), size of child at birth, birth order, twin status, and sex of child. The other variables were sex of household head, community literacy level, community socio-economic status, and place of residence. The variables were not determined a priori; instead, based on parsimony, theoretical relevance and practical significance with health seeking behaviour for childhood illnesses [11,20]. Marriage was recoded into “never married (0)”, “married (1)”, “cohabiting (2)”, “widowed (3)”, and “divorced (4)”. We recoded parity (birth order) as “one birth (1)”, “two births (2)”, “three births (3)”, and “four or more births (4)”; religion as “Christianity (1)”, “Islam (2)”, “Traditionalist (3)”, and “no religion (4)”; size of child at birth as “larger than average”, “average”, and “smaller than average”; and twin status as “single birth” and “multiple birth”. Exposure to media was coded as yes and no, signifying whether a woman reads newspaper, listens to radio or watches television or not.

### Statistical analyses

The data were analysed with Stata version 14.2. The analyses were done in three steps. The first step was the computation of the prevalence of women’s health seeking behaviour for childhood illnesses in Burundi. The second step was a bivariate analysis using Pearson’s chi-square test of independence that calculated the prevalence and proportions of health seeking behaviour for childhood illnesses across the independent variables with their significance levels. Statistical significance was considered at a p-value less than 0.20. The choice of a P < 0.20, instead of the usual P ≤ 0.05, were influenced by two main reasons (a) the purpose of the bivariate analyses was to identify potential predictor variables for the multivariate analyses rather than testing hypothesis, and b) it would minimize the risk of excluding variables with a biological (theoretical) plausibility from the multivariate analyses due to reasons, including confounding [21,22]. However, the statistical significance of the results of the binary logistic regression analysis was determined at P ≤ 0.05, because of its common usage in medical research. Before conducting the binary logistic regression analysis, a multi-collinearity test was carried out among all the statistically significant variables to determine if there was evidence of multicollinearity between them. Using the variance inflation factor (VIF), the multicollinearity test showed that there was no evidence of collinearity among the explanatory variables (Mean VIF = 1.20, Max VIF = 1.53, Minimum = 1.03**)**. In all, two models were generated from the binary logistic regression analysis. The first model (Model I) was the bivariate analysis between each of the explanatory variables, covariates, and health seeking behavior for childhood illnesses. Model II which is the complete model, was a multivariate logistic regression analysis where all the variables were used against the dependent variable. The results of the regression analyses were presented as crude odds ratio (cOR) and adjusted odds ratio (aOR). A sample weight (v005/1,000,000) to correct for over and under sampling was applied and the “svy” command to account for the complex survey design and generalizability of the findings was also used. In this study, we relied on the Strengthening the Reporting of Observational Studies in Epidemiology’ (STROBE) statement in writing the manuscript [23].

### Ethical approval

This study used secondary data and therefore no further approval was required because the data is available in the public domain. However, the authors sought permission to use the data by applying to MEASURE DHS and obtained approval to use the data.

## Results

### Prevalence of healthcare seeking for childhood illnesses in Burundi

**Fig 2:**
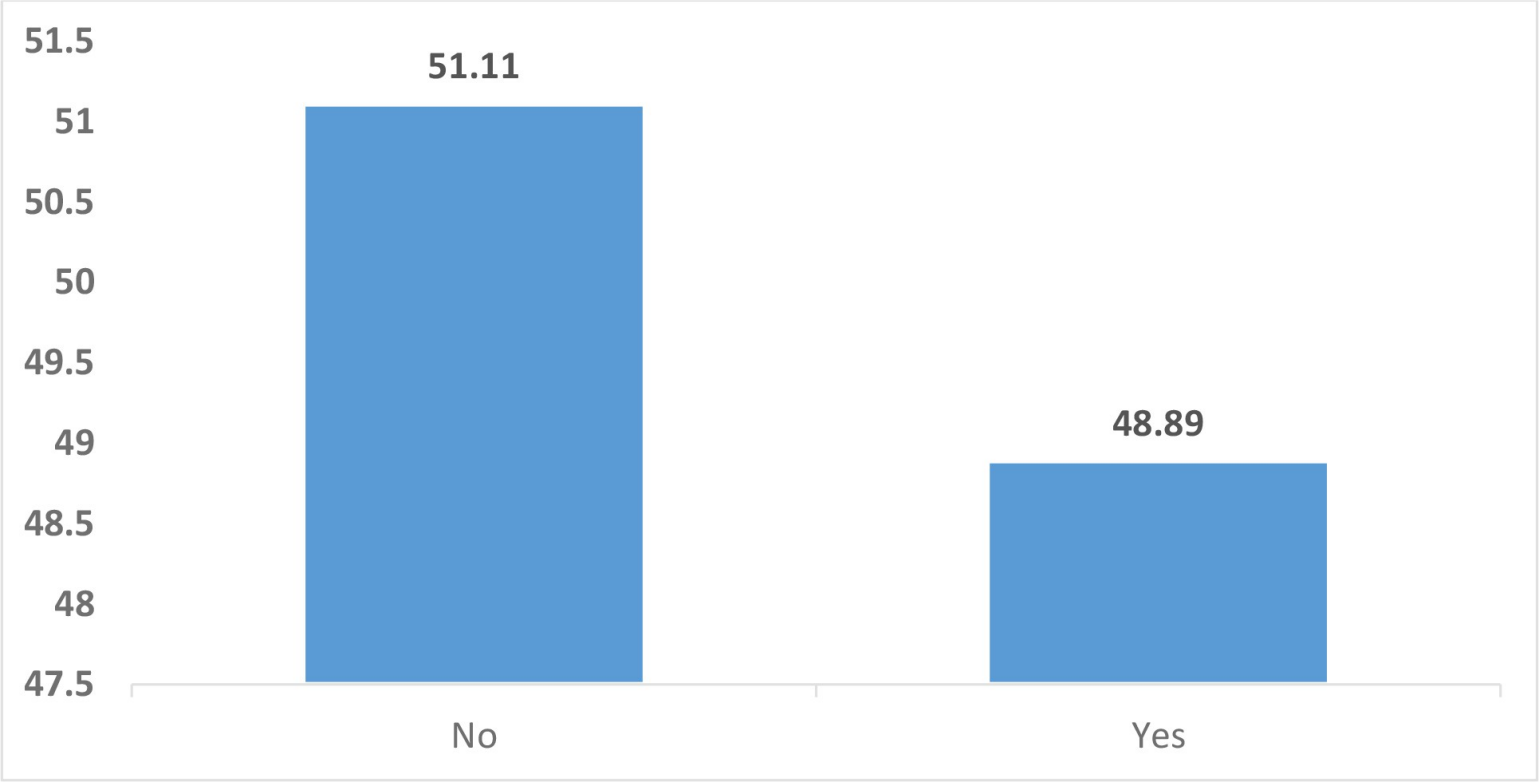
Prevalence of healthcare seeking for childhood illnesses in Burundi.

Fig 2 displays results from the study on the prevalence of healthcare seeking for childhood illnesses in Burundi. Overall, less than 50% of children in Burundi who were ill two weeks before the survey obtained healthcare.

#### Healthcare seeking behavior for childhood illness across independent variables

Table 1 shows results of the distribution of barriers to healthcare, control variables, and healthcare seeking for childhood illnesses among childbearing women in Burundi. At p<0.20, all the explanatory variables showed significant associations with healthcare seeking for childhood illnesses. Specifically, 65.8% of children of mothers who indicated that getting permission for medical care for self was not a big problem got healthcare for childhood illnesses while 59.8% of those whose mothers indicated that getting permission for medical care for self was a big problem did not get healthcare for childhood illnesses. Again, 70.8% of children of mothers who indicated that getting money for medical care for self was not a big problem got healthcare for childhood illnesses while 62.8% of those whose mothers indicated that getting money for medical care for self was a big problem did not get healthcare for childhood illnesses. Moreover, 66.7% of children of mothers indicated that distance to facility for medical care for self was not a big problem got healthcare for childhood illnesses while 62.9% of those whose mothers indicated that distance to facility for medical care for self was a big problem did not get healthcare for childhood illnesses. Finally, 67.1% of children of mothers who indicated that wanting to go for medical care alone was not a big problem got healthcare for childhood illnesses while 58.1% of those whose mothers indicated that wanting to go for medical care alone was a big problem got healthcare for childhood illnesses. In terms of the control variables, parity, religion, size of the child at birth, twin status, community literacy level, community socio-economic status and place of residence showed significant associations with healthcare seeking for childhood illnesses.

**Table 1:** Healthcare seeking behavior for childhood illness across independent variables.

Variable	Weighted N	Weighted %	Health seeking behavior	χ2 (p-value)
**Getting permission for medical care for self**				2.9 (0.090)
Big problem	142	6.5	59.8	
Not a big problem	2031	93.5	65.8	
**Getting money for medical care for self**				13.2 (<0.001)
Big problem	1494	68.7	62.9	
Not a big problem	679	31.3	70.8	
**Distance to facility for medical care for self**				4.4 (0.04)
Big problem	743	34.2	62.9	
Not a big problem	1430	65.8	66.7	
**Wanting to go for medical care alone**				15.5 (<0.001)
Big problem	413	19.0	58.1	
Not a big problem	1760	81.0	67.1	
**Age**				14.2 (0.028)
15–19	51	2.4	49.1	
20–24	488	22.5	65.7	
25–29	634	29.2	68.3	
30–34	482	22.2	67.3	
35–39	309	14.2	61.5	
40–44	162	7.4	65.6	
45–49	46	2.1	46.1	
**Marital status**				3.4 (0.500)
Not married	62	2.8	53.6	
Married	1282	59.0	66.3	
Cohabiting	614	28.2	66.6	
Widowed	39	1.8	56.9	
Divorced	176	8.1	60.7	
**Parity**				7.4 (0.061)
One birth	283	13.0	59.7	
Two births	403	18.6	65.7	
Three births	400	18.4	67.8	
Four or more births	1087	50.0	65.9	
**Employment status**				0.08 (0.783)
Not working	108	5.0	64.6	
Working	2065	95.0	65.4	
**Religion**				4.9 (0.084)
Christianity	2028	93.3	65.2	
Islam	63	2.9	78.9	
No religion	82	3.8	60.5	
**Exposure to mass media**				1.1 (0.297)
No	1683	77.5	65.7	
Yes	490	22.5	64.6	
**Sex of household head**				1.5 (0.225)
Male	1714	78.9	66.7	
Female	459	21.1	60.7	
**Size of child at birth**				7.4 (0.025)
Larger than average	737	33.9	61.9	
Average	991	45.6	68.1	
Smaller than average	445	20.5	65.2	
**Birth order**				1.8 (0.410)
First	431	19.9	65.3	
2–4	1024	47.1	67.1	
5+	718	33.0	63.0	
**Twin status**				21.1 (<0.001)
Single birth	2046	94.2	64.1	
Multiple birth	127	5.8	85.7	
**Sex of child**				0.8 (0.373)
Male	1150	52.9	66.1	
Female	1023	47.1	64.6	
**Community literacy level**				3.9 (0.146)
Low	796	36.6	65.1	
Medium	799	36.8	64.3	
High	578	26.6	67.4	
**Community socio-economic status**				4.1 (0.042)
Low	1537	70.7	64.5	
High	636	29.3	67.6	
**Place of residence**				4.9 (0.027)
Urban	129	5.9	68.1	
Rural	2044	94.1	65.2	

#### Binary logistic regression results on the predictors of healthcare seeking for childhood illnesses in Burundi

Table 2 shows the results on factors associated with healthcare seeking for childhood illnesses among childbearing women in Burundi. In the fully adjusted model, we found that children of mothers who perceived getting money for medical care for self as a big problem [aOR = 0.75; CI = 0.60–0.93] and considered going for medical care alone as a big problem [aOR = 0.71; CI = 0.55–0.91] had lower odds of getting healthcare, compared to those of mothers who considered these indicators as not a big problem. The results also showed that children of mothers who had three [aOR = 1.48; 1.02–2.15] and four [aOR = 1.62; 1.10–2.39], children were more likely to get healthcare for childhood illnesses compared to those whose mothers had one child. Children of mothers with single birth children were less likely to get healthcare compared to those whose mothers had multiple births.

**Table 2:** Binary logistic regression results on the predictors of healthcare seeking for childhood illnesses in Burundi.

Variables	Model I cOR [95% CI		Model I aOR [95%CI]
**Getting permission for medical care for self**			
Big problem	0.74 (0.53–1.05)		0.93 (0.65–1.35)
Not a big problem	Reference (1.0)		Reference (1.0)
**Getting money for medical care for self**			
Big problem	0.70*** (0.57–0.85)		0.75*** (0.61–0.93)
Not a big problem	Reference (1.0)		Reference (1.0)
**Distance to facility for medical care for self**			
Big problem	0.82* (0.69–0.99)		1.02 (0.81–1.27)
Not a big problem	Reference (1.0)		Reference (1.0)
**Wanting to go for medical care alone**			
Big problem	0.65*** (0.52–0.81)		0.71*** (0.55–0.91)
Not a big problem	Reference (1.0)		Reference (1.0)
**Age**			
15–19	Reference (1.0)		Reference (1.0)
20–24	2.10* (1.18–3.74)		1.71 (0.93–3.12)
25–29	2.24** (1.27–3.96)		1.56 (0.83–2.92)
30–34	2.02* (1.14–3.60)		1.22 (0.63–2.38)
35–39	1.91* (1.06–3.45)		1.12 (0.56–2.24)
40–44	1.89* (1.01–3.53)		1.13 (0.55–2.33)
45–49	1.04 (0.47–2.28)		0.62 (0.26–1.50)
**Parity**			
One birth	Reference (1.0)		Reference (1.0)
Two births	1.25 (0.91–1.71)		1.16 (0.82–1.64)
Three births	1.52* (1.10–2.10)		1.48* (1.02–2.15)
Four or more births	1.37* (1.04–1.80)		1.62* (1.10–2.39)
**Religion**			
Christianity	1.07 (0.66–1.71)		0.95 (0.57–1.57)
Islam	1.96 (0.96–4.00)		1.46 (0.68–3.12)
No religion	Reference (1.0)		Reference (1.0)
**Size of child at birth**			
Larger than average	0.84 (0.65–1.07)		0.89 (0.69–1.16)
Average	1.10 (0.87–1.39)		1.17 (0.92–1.50)
Smaller than average	Reference (1.0)		Reference (1.0)
**Twin status**			
Single birth	0.34*** (0.21–0.55)		0.38*** (0.23–0.63)
Multiple birth	Reference (1.0)		Reference (1.0)
**Community literacy level**			
Low	Reference (1.0)		Reference (1.0)
Medium	0.91 (0.74–1.11)		0.88 (0.71–1.10)
High	1.14 (0.91–1.42)		1.04 (0.82–1.33)
**Community socio-economic status**			
Low	Reference (1.0)		Reference (1.0)
High	1.22* (1.01–1.49)		1.16 (0.93–1.44)
**Residence**			
Urban	Reference (1.0)		Reference (1.0)
Rural	0.72* (0.53–0.96)		0.82 (0.59–1.15)

Exponentiated coefficients; 95% confidence intervals in brackets; cOR = Crude odds ratio; aOR = Adjusted odds ratio.

* *p* < 0.05

** *p* < 0.01

*** *p* < 0.001.

## Discussion

The current study investigated the barriers to healthcare access and health seeking for childhood illnesses in Burundi. We found that less than 50% of children in Burundi who were ill two weeks before the survey obtained healthcare. The prevalence of healthcare seeking for childhood illness was lower than what was recorded in Gabon with 75.0% [24]. Disparity in socio-cultural factors could influence the difference in the prevalence of healthcare seeking for childhood illness among countries. Again, the disparities in the findings could also be due to differences in sample size.

Children of mothers who had a big problem getting money for medical care for self were less likely to get healthcare for their illnesses as compared to those of mothers who had no problem getting money for healthcare. This finding supports other studies conducted in Ethiopia [25] and Kenya, Niger and Nigeria [26] that financial problem is a barrier to healthcare seeking behaviour for childhood illness. The current finding lends support to other array of research that have established strong connection between wealth and various health outcomes. Women from the poorest wealth quintile or poorest households usually demonstrate considerable poorer maternal health outcomes in low- and middle–income countries [27–30]. Such women might prioritize and use their less finances on other pressing needs such as food for the family at the expense of accessing healthcare for their children. For countries within SSA that have health insurance schemes such as Ethiopia, Ghana, Kenya and Rwanda [31], the barrier to healthcare seeking for childhood illnesses may not be financial matters but the involvement of other associated costs such as the cost of transportation to the health facilities [32]. Leveraging the free health care policy in Burundi targeting pregnant women and children under-five, the Health Sector Development Support Project introduced in 2006 seeks to increase health service utilization among pregnant women, children under-five and couples of reproductive age [33]. The government of Burundi should strengthen the zhealth system for maternal and child health and make additional investments in infrastructure and equipment to increase the utilization of healthcare for childhood illnesses.

Children of mothers who considered going for medical care alone as a big problem had lower odds of getting healthcare for their illnesses. The plausible explanation for this finding could be that men play a paramount role in determining the health needs of the family in SSA [34]. This is because men control most of the resources in marriages and hence, they decide where and when women should seek healthcare for childhood illnesses [35]. In view of this, women consider going for medical care alone as a big problem and this has a serious impact on health in particular on the women and children. Fenny, Yates and Thompson [31] also reported that some countries within the sub-Saharan African region with health insurance schemes, the barrier to seek for healthcare for their children could be associated with indirect costs of healthcare such as transportation cost to health facilities.

We found that children of mothers with high parity and those who had twins were more likely to get healthcare for childhood illnesses. This finding contradicts the findings of Abegaz, Berhe and Gebretekle [12] in Ethiopia who reported that women with low parity were more likely to seek healthcare for the sick child as compared to women with high parity. Abegaz, Berhe and Gebretekle [12] further elaborated that mothers’ high workload due to large family size could bring about giving lesser attention to the sick child.

### Strengths and limitations

The study has some strengths and limitations. The secondary data used for the analysis was drawn from a nationally representative survey collected by employing a two-stage stratified sampling technique. The analyses with discrete regression models using confidence intervals helps to determine the level of precision, provide more credible findings. However, the cross-sectional nature of the survey limits causal inferences and noted findings should be interpreted with caution. Finally, we hypothesized that personal health seeking behavior and mothers’ health seeking behaviour for a child will be similar. However, this may not always be true and limits the generalisability of the findings.

#### Practical implications

Burundi’s existing governmental initiatives could target women in poor households in order to remove the financial barriers they face when accessing healthcare. For example, lessening and/ or removal of all forms of user fees in government health care facilities would be productive towards the protection of households from expensive costs of illness.

## Conclusion

Findings of the low prevalence of healthcare for childhood illnesses in Burundi suggest the need for government and non-governmental organizations to strengthen women’s healthcare accessibility for child healthcare services and health seeking behaviours. The Burundian government through multi-sectoral partnership should strengthen health system for maternal health and address structural determinants of women’s health by creating favourable conditions to improve the status of women and foster their overall socioeconomic well-being. Free child healthcare policies in Burundi should be strengthened to enhance the utilization of child healthcare services in Burundi.

## References

[1] United Nations Inter-agency Group for Child Mortality Estimation. *Levels & trends in child mortality report 2019*. UN IGME. 2019. Retrieved from: https://childmortality.org/wp-content/uploads/2019/10/UN-IGME-Child-Mortality-Report-2019.pdf.

[2] WHO. *Children*: *reducing mortality*.2019. Retrieved from:https://www.who.int/news-room/fact-sheets/detail/children-reducing-mortality.

[3] Moise I. K. Causes of Morbidity and Mortality among Neonates and Children in Post-Conflict Burundi: A Cross-Sectional Retrospective Study. *Children*:2018:5(9): 125. doi: 10.3390/children5090125 30205549 PMC6162533

[4] Fullman N., Yearwood J., Abay S. M., Abbafati C., Abd-Allah F., Abdela J., et al. Measuring performance on the Healthcare Access and Quality Index for 195 countries and territories and selected subnational locations: a systematic analysis from the Global Burden of Disease Study 2016. *The Lancet*:2018:391(10136):2236–2271. doi: 10.1016/S0140-6736(18)30994-2 29893224 PMC5986687

[5] Rutherford M. E., Mulholland K., & Hill P. C. How access to health care relates to under‐five mortality in sub-Saharan Africa: systematic review. *Tropical Medicine & International Health*:2010:15(5): 508–519. doi: 10.1111/j.1365-3156.2010.02497.x 20345556

[6] Qamar F. N., Zaman U., Quadri F., Khan A., Shaikh B. T., Azam I., et al. Predictors of diarrheal mortality and patterns of caregiver health seeking behavior in Karachi, Pakistan. *Journal of Global Health*:2016:6(2). 10.7189/jogh.6.020406 PMC5012233 27606059

[7] Wambui W. M., Kimani S., & Odhiambo E. Determinants of health seeking behavior among caregivers of infants admitted with acute childhood illnesses at Kenyatta National Hospital, Nairobi, Kenya. *International Journal of Pediatrics*:2018. doi: 10.1155/2018/5190287 30643520 PMC6311279

[8] Adekanmbi V. T., Adedokun S. T., Taylor-Phillips S., Uthman O. A., & Clarke A. Predictors of differences in health services utilization for children in Nigerian communities. *Preventive Medicine*:2017:96: 67–72. doi: 10.1016/j.ypmed.2016.12.035 28040520 PMC5340469

[9] Adane M., Mengistie B., Mulat W., Kloos H., & Medhin G. Utilization of health facilities and predictors of health-seeking behavior for under-five children with acute diarrhea in slums of Addis Ababa, Ethiopia: a community-based cross-sectional study. *Journal of Health*, *Population and Nutrition*:2017:36(1):9. 28376916 10.1186/s41043-017-0085-1 PMC5381138

[10] Akinyemi J. O., Banda P., De Wet N., Akosile A. E., & Odimegwu C. O. Household relationships and healthcare seeking behaviour for common childhood illnesses in sub-Saharan Africa: a cross-national mixed effects analysis. *BMC Health Services Research*:2019:19(1): 308. doi: 10.1186/s12913-019-4142-x 31088474 PMC6518738

[11] Lungu E. A., Darker C., & Biesma R. Determinants of healthcare seeking for childhood illnesses among caregivers of under-five children in urban slums in Malawi: a population-based cross-sectional study. *BMC Pediatrics*:2020:20(1):20. Available at: doi: 10.1186/s12887-020-1913-9 31952484 PMC6966883

[12] Abegaz N. T., Berhe H., & Gebretekle G. B. Mothers/caregivers healthcare seeking behavior towards childhood illness in selected health centers in Addis Ababa, Ethiopia: a facility-based cross-sectional study. *BMC Pediatrics*:2019:19(1): 220. doi: 10.1186/s12887-019-1588-2 31269920 PMC6607537

[13] Simieneh M. M., Mengistu M. Y., Gelagay A. A., & Gebeyehu M. T. Mothers’ health care seeking behavior and associated factors for common childhood illnesses, Northwest Ethiopia: community based cross-sectional study. *BMC Health Services Research*:2019:19(1):59. doi: 10.1186/s12913-019-3897-4 30674309 PMC6343298

[14] Tette E., Nuertey B. D., Azusong E. A., & Gandau N. B. The Profile, Health Seeking Behavior, Referral Patterns, and Outcome of Outborn Neonates Admitted to a District and Regional Hospital in the Upper West Region of Ghana: A Cross-Sectional Study. *Children*:2020:7(2):15. 10.3390/children7020015 PMC7072572 32085390

[15] Ayalneh A. A., Fetene D. M., & Lee T. J. Inequalities in health care utilization for common childhood illnesses in Ethiopia: evidence from the 2011 Ethiopian Demographic and Health Survey. *International Journal for Equity In Health*:2017:16(1):67. doi: 10.1186/s12939-017-0561-7 28431502 PMC5399816

[16] Victora C. G., Barros A. J., Blumenberg C., Costa J. C., Vidaletti L. P., Wehrmeister F. C., et al. Association between ethnicity and under-5 mortality: analysis of data from demographic surveys from 36 low-income and middle-income countries. *The Lancet Global Health*:2020:8(3):e352–e361. doi: 10.1016/S2214-109X(20)30025-5 32087172 PMC7034191

[17] Awoke W. Prevalence of childhood illness and mothers’/caregivers’ care seeking behaviour in Bahir Dar, Ethiopia: a descriptive community based cross sectional study. *Open Journal of Preventive Medicine*:2013: 3:155–9.

[18] Corsi DJ, Neuman M, Finlay JE, Subramanian SV. Demographic and health surveys: a profile. International journal of epidemiology. 2012 Dec 1;41(6):1602–13. doi: 10.1093/ije/dys184 23148108

[19] Aliaga A, Ruilin R. Cluster optimal sample size for demographic and health surveys. In7th International Conference on Teaching Statistics–ICOTS 2006 Jul (Vol. 7, pp. 2–7).

[20] Taffa N, Chepngeno G. Determinants of health care seeking for childhood illnesses in Nairobi slums. Tropical Medicine & International Health. 2005 Mar;10(3):240–5. doi: 10.1111/j.1365-3156.2004.01381.x 15730508

[21] Bursac Z, Gauss CH, Williams DK, Hosmer DW. Purposeful selection of variables in logistic regression. Source code for biology and medicine. 2008;3:17. doi: 10.1186/1751-0473-3-17 19087314 PMC2633005

[22] Victora CG, Huttly SR, Fuchs SC, Olinto MT. The role of conceptual frameworks in epidemiological analysis: a hierarchical approach. Int J Epidemiol. 1997;26:224–7. doi: 10.1093/ije/26.1.224 9126524

[23] Von Elm E., Altman D. G., Egger M., Pocock S. J., Gøtzsche P. C., & Vandenbroucke J. P. The Strengthening the Reporting of Observational Studies in Epidemiology (STROBE) statement: guidelines for reporting observational studies. *Annals of Internal Medicine*:2007:147(8):573–577. doi: 10.7326/0003-4819-147-8-200710160-00010 17938396

[24] Direction Générale de la Statistique (DGS) et ICF International. 2013. Enquête Démographique et de Santé du Gabon 2012. Calverton, Maryland, et Libreville, Gabon: DGS et ICF International.

[25] Assefa T, Belachew T, Tegegn A, Deribew, A. Mothers’ Health Care Seeking Behavior for Childhood Illnesses in Derra District, Northshoa Zone, Oromia Regional State, Ethiopia. *Ethiopian Journal of Health Science*:2008:18(3):87–94.

[26] Bedford KJ, Sharkey AB. Local Barriers and Solutions to Improve Care-Seeking for Childhood Pneumonia, Diarrhoea and Malaria in Kenya, Nigeria and Niger: A Qualitative Study. *PLoS ONE*:2014:9(6):e100038. doi: 10.1371/journal.pone.0100038 24971642 PMC4074042

[27] Amooti-Kaguna B, Nuwaha F. Factors influencing choice of delivery sites in Rakai district of Uganda. *Soc Sci Med*: 2000:50(2):203–213. doi: 10.1016/s0277-9536(99)00275-0 10619690

[28] Magadi MA, Madise NJ, Rodrigues RN. Frequency and timing of antenatal care in Kenya: explaining the variations between women of different communities. *Soc Sci Med*:2000:51(4):551–561. doi: 10.1016/s0277-9536(99)00495-5 10868670

[29] Onah HE, Ikeako LC, Iloabachie GC. Factors associated with the use of maternity services in Enugu, southeastern Nigeria. *Soc Sci Med*:2006:63(7):1870–1878. doi: 10.1016/j.socscimed.2006.04.019 16766107

[30] Zulu E, Dodoo FN, Ezeh CA. Sexual risk-taking in the slums of Nairobi, Kenya, 1993–98. *Popul Stud*:2002:56:311–323. 10.1080/00324720215933 12553329

[31] Fenny AO, Yates R, Thompson R. Social health insurance schemes in Africa leave out the poor. *Int Health*:2018:10: 1–3. doi: 10.1093/inthealth/ihx046 29325056

[32] Anafi P, Mprah W. K., Jackson A. M., Jacobson J. J., Torres C. M., Crow B. M., et al. Implementation of Fee-Free Maternal Health-Care Policy in Ghana: Perspectives of Users of Antenatal and Delivery Care Services from Public Health-Care Facilities in Accra. *International Quarterly of Community Health Education*:2018:38(4):259–267. doi: 10.1177/0272684X18763378 29523057

[33] The World Bank Group. RBF Health. 2014. www.rbfhealth.org.

[34] Shaikh BT, Hatcher J. Health seeking behaviour and health service utilization in Pakistan: challenging the policy makers. Journal of public health. 2005 Mar 1;27(1):49–54. doi: 10.1093/pubmed/fdh207 15590705

[35] Rani M, Bonu S. Rural Indian women’s care‐seeking behavior and choice of provider for gynecological symptoms. Studies in Family Planning. 2003 Sep;34(3):173–85. doi: 10.1111/j.1728-4465.2003.00173.x . 14558320

